# The effect of Pimpinella Anisum herbal tea on human milk volume and weight gain in the preterm infant: a randomized controlled clinical trial

**DOI:** 10.1186/s12906-023-03848-6

**Published:** 2023-01-21

**Authors:** Sona Khalili, Leila Amiri-Farahani, Shima Haghani, Arash Bordbar, Asie Shojaii, Sally Pezaro

**Affiliations:** 1 Department of Reproductive Health and Midwifery, School of Nursing and Midwifery, Iran University of Medical Sciences, Tehran, Iran; 2 Department of Reproductive Health and Midwifery, Nursing and Midwifery Care Research Center, School of Nursing and Midwifery, Iran University of Medical Sciences, Tehran, Iran; 3 Department of Biostatistics, Nursing Care Research Center, Iran University of Medical Sciences, Tehran, Iran; 4 Shahid Akbarabadi Clinical Research Development Unit (ShACRDU), Iran University of Medical Sciences (IUMS), Tehran, Iran; 5 School of Persian Medicine, Research Institute for Islamic and Complementary Medicine, Iran University of Medical Sciences, Tehran, Iran; 6 The Centre for Healthcare research, Coventry University, Coventry, United Kingdom

**Keywords:** Pimpinella anisum, Breast milk production, Milk volume, Preterm infants, Galactagogue herbal tea, Newborn weight, Lactation, Human milk, Breastfeeding

## Abstract

**Background and aims:**

Human milk supports pre-term infants to thrive. Yet human milk production can be inhibited when infants are born prematurely. Pimpinella Anisum has been evidenced to increase milk production and infant weight gain in previous animal studies. The present study aimed to determine the effect of Pimpinella Anisum herbal tea on human milk volume and preterm infant weight in human populations for the first time.

**Methods:**

Human milk supports pre-term infants to thrive. Yet human milk production can be inhibited when infants are born prematurely. Pimpinella Anisum has been evidenced to increase milk production and infant weight gain in previous animal studies. The present study aimed to determine the effect of Pimpinella Anisum herbal tea on human milk volume and preterm infant weight in human populations for the first time.

**Results:**

There was a statistically significant difference in terms of milk volume in the first, third, fourth, fifth, sixth and seventh days between the three groups of intervention, placebo, and control (*p* < 0.05). On the first day, the mean volume of pumped milk in the intervention group was significantly higher than the control group (*p* = 0.008). On the second day, there was no statistically significant difference between groups. On the third, fourth, fifth, sixth and seventh days, the mean volume of pumped milk in the intervention group was significantly higher than the placebo and control groups (*p* < 0.05). There was no statistically significant difference in terms of preterm infant weight on days 0, 3 and 7 between the three groups.

**Conclusion:**

The use of Pimpinella Anisum or ‘Anise’ tea can increase the volume of human milk and since no specific side effects have been reported in its use, it may be incorporated easily, cheaply, and effectively in practice where appropriate to the benefit of preterm infant nutrition worldwide.

## Introduction

In 2018 only 42% of infants started breastfeeding in the first hour of life [1]. Due to its numerous health benefits, one of the global goals of infant nutrition by 2025 is that at least 50% of infants under the age of 6 months be exclusively breastfed [2]. In Iran, the results of a 2018 systematic review and meta-analysis reported the overall prevalence of exclusive breastfeeding to be 53% [3]. This is encouraging, as human milk feeding is beneficial for all infants, including those born preterm [4], which are 40 times more likely to die than infants born at full term [5], and yet may be saved from conditions such as necrotizing enterocolitis by being exclusively breast fed [6]. Nonetheless, when preterm infants are admitted to neonatal intensive care units, the maintenance of human milk production, and the rate of successful breastfeeding can be lower than it would be otherwise [7, 8]. Therefore, breastfeeding support, protection and promotion are of high importance in this context [9].

The incomplete physical development which occurs in premature infants may lead to severe respiratory distress, hypoglycemia, and hypothermia, requiring the infant to be admitted to intensive care, and thus delay the onset of breastfeeding alongside parental separation [10]. In addition, neuralgia affects the tone of the oral system, causing poor sucking-swallowing coordination in the preterm infant [11]. This in turn means that the breast may not be emptied of milk effectively, which in turn can lead to reduced milk supply and even reduced milk production [12]. With inadequate milk consumption and reduced milk supply, nutrition-related deaths, diarrhea, stunted growth, and hyperbilirubinemia may occur, leading to neonatal rehospitalization [13]. Numerous studies have explored the reasons why exclusive breastfeeding may be discontinued [14–17]. For those who choose to breastfeed premature infants, the presence of excitement, fatigue, and psychological stress and illness, along with the inadequate emptying of milk may also prove inhibitive [18, 19]. Thus, to maximise the benefits of breastfeeding, it will be important to explore new ways in which to increase human milk volume to the benefit of newborns and families worldwide.

Strategies to overcome the challenges associated with breastfeeding include the use of nipple shields, cup feeding, complementary milk, and syringe feeding [12]. Additionally, in the absence of breastfeeding counseling and non-pharmacological strategies, researchers' attention to the use of chemical and herbal milk supplements has increased [14, 20]. In many parts of the world, herbal medicines have been used for the augmentation of human milk during postpartum period [21, 22]. Pimpinella Anisum or ‘Aniseed/Anise’ in traditional medicine is already established to have many therapeutic benefits in humans including being an antioxidant, antibacterial, antifungal, anticonvulsant, anti-inflammatory, analgesic, gastro-protective, antidiabetic, and antiviral [23, 24]. In terms of chemical composition, approximately 90–80% of Pimpinella Anisum is Anethole [24], which is known to lead to increased prolactin stimulation and milk production in animals [25, 26]. A recent review of the literature in this field revealed only further studies including animals, and none thus far including human participants [27]. This provides a unique opportunity to conduct founding research into the effects of Pimpinella Anisum on human milk volume, and subsequent thriving of the premature infant as it is reasonable to assume that such effects seen in animal populations may be similar in humans.

In justifying the testing of Pimpinella Anisum in humans, we first considered safety in high regard. Indeed, anise is one of the drugs approved by the Food and Drug Administration of Iran with the registration number 20885/56 produced by Sabz Andish Parnian Pharmaceutical Company [28]. Moreover, citing paragraph 4, Article 3 of the description of the duties of the midwife, the resolution dated 2009, which is related to the care of the infant [29] as well as Article 6; the midwife is permitted to use medicinal plants licensed by the Ministry of Health, Treatment and Medical Education in the treatment of gynecological and obstetric problems. Moreover, Studies conducted thus far exploring Pimpinella Anisum use in animal populations have not identified any adverse side effects [25, 30, 31]. Considering the above, the present study aimed to determine the effect of Pimpinella Anisum herbal tea on human milk volume and preterm infant weight in human populations for the first time.

## Methods

### Trial design and participants

Our study was conducted as a parallel randomised clinical trial with intervention, control, and placebo groups. Reporting of this study has been done in accordance with the Consolidation Standards of Reporting Trials (CONSORT) statement. It was funded by Iran University of Medical Sciences and conducted in July 2021. The study’s protocol was registered in the Clinical Trial Registration Center on 14 March 2021. Code: IRCT20180427039436N9. Our study population included breast feeders and premature infants whose gestational age was less than 32 weeks who were hospitalized in the neonatal intensive care unit of Akbarabadi Hospital (referral and educational hospital) in Tehran, Iran.

#### Maternal inclusion criteria

Participants were included if they had a desire to exclusively breastfeed and pump human milk using an electronic pump from the third day following birth. Included participants were also required to be literate and at least 18 years of age.

#### Infant inclusion criteria

Infants were required to have been born preterm (at less than 32 weeks of gestation), and admitted to the intensive care unit, where such infants are typically fed by orogastric or nasogastric tube.

#### Maternal exclusion criteria

Participants were excluded if they were engaged in smoking, alcohol, and/or drug use, suffering from infectious diseases transmitted through human milk feeding (e.g., AIDS), had active pulmonary tuberculosis, had a history of infertility, were taking anticoagulants which are contraindicated with the use of Anise [32], had a history of cancer estrogen-dependent diseases (e.g., uterine cancer, breast cancer, endometrial hyperplasia) and/or had mastectomy, nipple fractures, breast abscesses and/or infectious mastitis.

#### Infant's exclusion criteria

Infants were excluded if they had congenital anomalies (e.g., cleft lip and palate), and/or were born as part of a multiple birth.

#### Withdrawal criteria

Participants were able to withdraw at any time without giving a reason. Whilst the supplementation of iron and multivitamins are recommended from the 16th week of pregnancy until three months after giving birth, our withdrawal criteria included cases in which there was use of any additional galactagogues (human milk inducers/enhancers). Participants were similarly withdrawn if they were consuming less than 10% of Pimpinella Anisum herbal tea, along with cases of allergic reaction and intolerance.

### Data collection/recruitment

Convenience sampling was used to recruit eligible participants over a period of 6 months. Those who met the eligibility criteria were given participant information and those who subsequently agreed to take part in the study were invited to complete informed consent forms and enrolled.

Data collection was carried out between July 2021 and November 2021. Eligible participants were assigned to three groups: (1) intervention, (2) placebo and (3) control using the block randomization method (https://www.sealedenvelope.com) and followed for a period of 7 days. To determine the sequence of participants’ allocation based on the block randomization method, it is necessary to ascertain the total sample size, the number of groups and the number of group repetitions in each block (considered equal). In the current study, the size of each block was twice the number of groups (six groups in each block). The inclusion of a control group meant that it was not possible to assure blindness. Therefore, a randomization code was generated for each participant using a sealed envelope website. An epidemiologist, who was not part of the study assimilated the randomization list. For allocation concealment, the assignment list remained with the epidemiologist.

Human milk volume was recorded as one of the main outcomes of the study by participants in a daily information recorder form. The infant's weight was also measured and recorded by the lead researcher. Statistical analysis was performed by a statistician who also did not know the content provided for the study groups and their allocation.

### Description of intervention

Participants in the intervention group received Pimpinella Anisum herbal tea (2 g of dried Anise plant plus 1 g of black tea). Those in the placebo group received black tea containing 3 g of dried black tea only. In both groups, tea was ingested 3 times [32–34] a day for a week at breakfast, lunch, and dinnertime. The intervention period was limited to 7 days, because typically after this time the preterm infant is fed human milk directly (rather than being tube fed), and thus the recording of milk production/volume becomes impossible. Uniformity and same-color packaging for herbal compounds was considered in both intervention and placebo groups. Both teas were prepared by the School of Persian Medicine in Iran University of Medical Sciences under the supervision of a specialized consultant of medicinal plants (PhD of Pharmacognosy) (A.SH.). Participants placed a tea bag in the cup, added 150 ml of boiling water, and sipped the tea after 10 min of infusion. Those in the control group did not receive any herbal or chemical composition of galactagogue. At the end of the intervention period, those in the control group were offered Pimpinella Anisum herbal tea also.

Participants in the intervention and placebo groups had no information about the type of herbal tea they were ingesting. To increase accuracy in relation to the intervention and placebo groups, the participants, the researcher, and statistical analyst did not know anything about the type of herbal tea ingested by the either the intervention or placebo groups.

Participants in all groups used the Medela electronic machine to pump their milk at least 6 times a day (3–4 hourly upon waking at approximately 8am) for a period of 7 days. Excess milk was stored in the milk bank of Akbarabadi Hospital.

### Assessment of trial variables

The variables of this study were measured as follows:

#### Demographics and baseline characteristics

Demographics and baseline data were related to both parents and the infant. Maternal data was collected in relation to age, body mass index, education level, employment status, whether the pregnancy had been planned (or not), number of previous births, mode of birth, chronic diseases, use of drugs during pregnancy and steroid use, as well as consumption of iron and other supplements. Paternal data collected included occupation type, level of education, and socio-economic status. Data collected in relation to the infant included birth weight, height, head circumference, gestational age at birth, neonatal age upon entry to the study, observed sex at birth, cause of hospitalization and type of serum injected into the infant (e.g., Dextrose water) along with the infant feeding method chosen. Maternal food consumption known to increase human milk production such as lettuce, basil leaves, dill, fenugreek, carrot juice, spinach, sesame, and fennel was also monitored. Data relating to the infant was extracted from medical records by the researcher. Maternal and paternal data was recorded and self-reported by the parents.

#### Daily information recorder form

The daily information form included space for data entry in relation to the frequency of pump use, time spent on pump, volume of milk produced at each pumping, time spent skin-to-skin with the baby, frequency of liquids and tea based on the number of glasses consumed during the day, and the amount of pumped milk at the end of each day. All participants pumping human milk in the study completed this form daily for 7 days.

#### Infant weight recorder form

The infant's weight was measured by a German Seca scale on day 0 (before intervention), day 3 and day 7 of the intervention period. Measurements were taken by the researcher during the morning shift prior to the first human milk feed and after the infant’s first bowel and urinary outputs. Values were recorded in the infant weight recorder form.

### Sample size

To determine the required sample size at 95% confidence level and 80% test power, assuming that the effect of Pimpinella Anisum on human milk volume in the intervention group should be 25 unit (d = 25) for the effect to be considered statistically significant compared with the control group, the sample size in each group was estimated to be at least 38 people using the sample size formula. The total number of participants in this study was therefore 145 individuals.

Based on the study by Turkyılmaz et al., (2011) the standard deviation of milk volume was estimated to be 53.5 and 12.9 in the intervention and control groups, respectively. Also, with regards to the weight of neonate, the sample size of 15 people in each group was determined [34], allowing for a drop-out rate of 20%.

$$n=\frac{({{z}_{1- {\alpha }\!\left/ \! {2}\right.}+{z}_{1-\beta })}^{2}\times ({s}_{1}^{2}+{s}_{2}^{2})}{{d}^{2}}$$

$${z}_{0.975}=1.96$$

$${z}_{0.8}=0.84$$

$$n=\frac{({1.96+0.84)}^{2}\times ({53.5}^{2}+{12.9}^{2})}{{25}^{2}}\approx 38$$

### Ethics

The protocol of the present study was approved by the Ethics Committee of Iran University of Medical Sciences with the ethics code: IR.IUMS.REC.1399.1417. All participants were fully informed about the objectives and process of the study and informed written consent was obtained from them. The information obtained during the study process remained completely confidential. All participants were informed that they could withdraw from the study at any time. No fees were paid for the study and all services were completely free. Only one code was assigned to the study, and all study data were entered into data systems anonymously. All participants were given the researcher's phone number at the beginning of the study, as participants were asked to inform the researcher of any side effects.

### Statistical analysis

One-way analysis of variance (ANOVA) was used to statistically analyse the data and to compare the groups in terms of quantitative variables such as maternal age, Body Mass Index (BMI), infants' gestational age at birth and age upon entry to the study, infant’s weight, height and head circumference, and maternal consumption of fluids and tea (glass/day). Fisher's exact test was used to compare the groups in terms of variables such as economic status, mode of birth, paternal education level, and type of serum injected into the infant (e.g., Dextrose water). Chi-squared test was used to compare the group in terms of maternal and paternal occupation, maternal education and number of previous births, infant’s observed sex at birth, whether pregnancy was planned or unplanned, chronic diseases, any drug use during pregnancy, maternal use of steroids, supplements and drugs, cause of infant hospitalization and chosen infant feeding method.

One-way ANOVA was used to compare the frequency of pumping, time spent on pump, time spent skin-to-skin with the infant, frequency of liquids and tea based on the number of glasses consumed during the day, amount of pumped milk at the end of each day, and infant weight in the groups on the first to seventh days after the start of the intervention. Analysis of variance with repeated measures was used for the comparison of the mean frequency of pump use, time spent on pump, time spent skin-to-skin with the infant, frequency of liquids and tea based on the number of glasses consumed during the day, amount of pumped milk at the end of each day, and infant weight over time (first to seventh days after the start of the interventions). Data were analysed by SPSS software version 22 (IBM Corp, US). In all tests, a p-value of < 0.05 was considered significant.

## Result

**Fig. 1:**
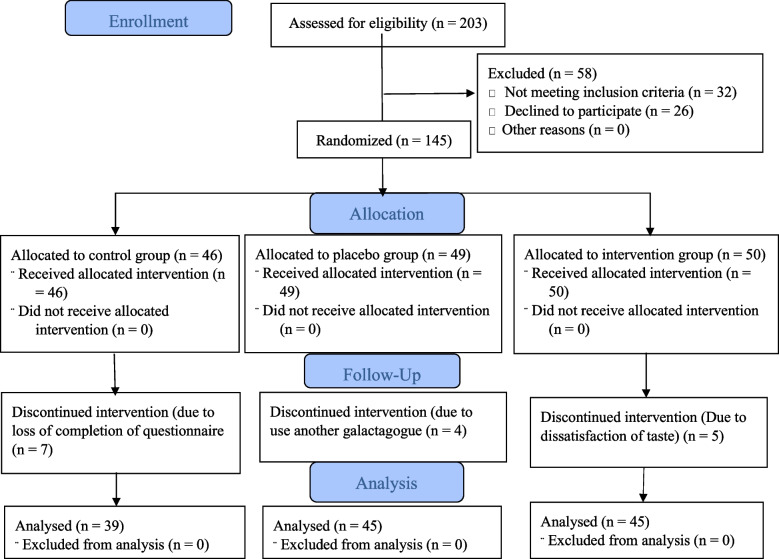
Enrolment of participants into three groups of intervention, placebo and control

Overall, 145 participants with preterm infants participated in the study (50 in the intervention group, 49 in the placebo group, and 46 in the control group). However, some (*n* = 5) were withdrawn due to being dissatisfied with the taste and thus non-compliant in ingesting the tea. Others (*n* = 4) were withdrawn either due to their use of other galactagogues or due to incomplete questionnaires (*n* = 7). Finally, 129 participants (45 in the intervention group, 45 in the placebo group, 39 in the control group) remained in the study and were statistically analyzed (Fig. 1). No adverse side effects were reported by participants.

The three groups did not demonstrate a statistically significant difference in terms of demographics, maternal, paternal, and infant baseline data or recorded daily information (Table 1). Yet in comparing the variables of the mean time spent on pump and the frequency of pumping in the three groups, a statistically significant difference was observed (Table 2). This difference is largely due to the enhancing effect of Anise, which required that these individuals spent more time and frequency pumping the larger amounts of milk produced. In terms of maternal consumption of foods with milk-enhancing properties in the three groups, there was no statistically significant difference observed in any of the seven days of the study (*P* ≥ 0.05).

**Table 1:** Demographics and baseline characteristics of study participants and comparisons between intervention group, placebo group and control group

Variables	Intervention group (*n* = 45)	Placebo group (*n* = 45)	Control group (*n* = 39)	*P*—value
**Maternal age (year), (Mean ± SD)**	30.72 ± 6.64	29.83 ± 6.99	32.29 ± 6.69	0.594
**Maternal BMI (kg/m**^**2**^**), (Mean ± SD)**	30.1 ± 24.79	30.1 ± 01.49	29.1 ± 81.85	0.479
**Maternal education, n (%)**				
Elementary	17 (34)	11 (16.3)	15 (32.6)	0.22
Secondary	11 (22)	21 (42.9)	17 (37)	
High school	14 (28)	13 (26.5)	10 (21.7)	
University	8 (16)	7 (14.3)	4 (8.7)	
**Maternal occupation, n (%)**				
Housewife	37 (74)	40 (81.6)	37 (80.4)	0.61
Employed	13 (26)	9 (18.4)	9 (19.6)	
**Pregnancy planned, n (%)**				
Yes	40 (80)	43 (87.8)	39 (84.8)	0.567
No	10 (20)	6 (12.2)	7 (15.2)	
**Number of previous births (Mean ± SD)**				
1	25 (50)	30 (61.2)	22 (7.8)	0.504
2	12 (24)	12 (24.5)	11 (23.9)	
> 3	13 (26)	7 (14.3)	13 (28.3)	
**Mode of birth, n (%)**				
Vaginal birth	42 (84)	45 (91.8)	44 (95.7)	0.151
Birth via caesarean section	8 (16)	4 (8.2)	2 (4.3)	
**Maternal chronic diseases, n (%)**				
Yes	14 (28)	7 (14.3)	13 (28.3)	0.108
No	36 (72)	42 (85.7)	40 (87)	
**Maternal drug use during pregnancy, n (%)**				
Yes	13 (26)	7 (14.3)	6 (13)	0.183
No	37 (74)	42 (85.7)	40 (87)	
**Maternal use of iron and complementary drugs, n (%)**				
Yes	35 (70)	37 (75.5)	37 (80.4)	0.496
No	15 (30)	12 (24.5)	9 (19.6)	
**Maternal use of steroids, n (%)**				
Yes	50 (100)	47 (95.9)	44 (95.7)	0.338
No	0 (0)	2 (4.1)	2 (4.3)	
**Paternal occupation, n (%)**				
Employee	7 (14)	10 (20.4)	12 (26.1)	0.418
Manual worker	9 (18)	13 (26.5)	10 (21.7)	
Self- employed	34 (68)	26 (53.1)	24 (52.2)	
**Paternal education, n (%)**				
Elementary	3 (6)	5 (10.2)	5 (10.9)	0.539
Secondary	22 (44)	22 (44.9)	16 (34.8)	
High school	19 (38)	17 (34.7)	14 (30.4)	
University	6 (12)	5 (10.2)	11 (23.9)	
**Economic status of the family, n (%)**				
Desirable	8 (16)	3 (6.1)	6 (13)	0.287
Relatively	41 (82)	41 (83.7)	37 (80.4)	
undesirable	1 (2)	5 (10.2)	3 (6.6)	
**Infant's weight, (gr)(Mean ± SD)**	1245.146 ± 22.84	1245.147 ± 42.08	1199.146 ± 45.8	0.22
**Infant's Height, (cm)(Mean ± SD)**	39.1 ± 5.58	39.1 ± 63.69	38.1 ± 97.48	0.111
**Head circumference of infant, (cm)(Mean ± SD)**	29.1 ± 5.97	29.2 ± 65.06	29.1 ± 6.86	0.327
**Gestational age at birth, (wk) (Mean ± SD)**	30.0 ± 1.83	30.0 ± 21.91	29.0 ± 92.96	0.229
**Neonatal age upon entry to the study, (wk) (Mean ± SD)**	8.67 ± 4.94	8.50 ± 5.26	8.73 ± 4.15	0.407
**Infant sex observed at birth, n (%)**				
Male	21 (42)	15 (30.6)	18 (39.1)	0.478
Female	29 (58)	34 (69.4)	28 (60.9)	
**Cause of infant hospitalization, n (%)**				
IUGR				
yes	8 (16)	7 (14.3)	14 (30.4)	0.099
no	42 (84)	42 (85.7)	32 (69.6)	
PDA				
yes	6 (12)	7 (14.3)	10 (21.7)	0.398
no	44 (88)	42 (85.7)	36 (78.3)	
IVH < 3				
yes	15 (30)	14 (28.6)	12 (26.1)	0.912
no	35 (70)	35 (71.4)	34 (73.9)	
**Type of infant feeding, n (%)**				
Nasogastric tube	12 (24)	12 (24.5)	15 (32.6)	0.571
Orogastric tube	38 (76)	37 (75.5)	31 (67.4)	
**Type of serum injected into the infant, n (%)**				
D.W 5%	35 (70)	35 (71.4)	30 (65.2)	0.968
D.W 7.5%	11 (22)	10 (20.4)	11 (23.9)	
D.W 10%	4 (8)	4 (8.2)	5 (8.2)	

*SD* standard deviation, *BMI* Body Mass Index, *IUGR* Intra Uterine Growth Restriction, *PDA* Patent Ductus Arteriosus, *IVH* Intra Ventricular Hemorrhage, *D.W* Dextrose water

**Table 2:** Recorded daily information and infant weight in three groups of study

Variables		Intervention group (*n* = 45)	Placebo group (*n* = 45)	Control group (*n* = 39)	*P* – value ^a, b, c, d^
Frequency of tea based on the number of glasses consumed during the day **(Mean ± SD)**	**1 day**	2.26 ± 0.85	1.97 ± 0.8	1.93 ± 0.99	0.148
	**2 day**	2.34 ± 0.98	2.2 ± 0.99	2.13 ± 1.18	0.614
	**3 day**	2.34 ± 1.13	2.22 ± 1.02	2.13 ± 1.1	0.642
	**4 day**	2.18 ± 1.06	2.02 ± 0.94	2.04 ± 1.22	0.731
	**5 day**	2.32 ± 0.99	2.24 ± 1.03	2.19 ± 1.22	0.852
	**6 day**	2.32 ± 1.03	2.31 ± 1.1	2.1 ± 1.23	0.592
	**7 day**	2.34 ± 1.06	2.26 ± 1.07	2.11 ± 1.23	0.593
Frequency of liquids based on the number of glasses consumed during the day **(Mean ± SD)**	**1 day**	1.4 ± 0.81	1.48 ± 0.76	1.28 ± 0.86	0.462
	**2 day**	1.2 ± 0.85	1.48 ± 0.98	1.17 ± 0.85	0.162
	**3 day**	1.06 ± 0.86	1.22 ± 0.87	1.3 ± 1.01	0.41
	**4 day**	0.98 ± 0.71	1.22 ± 0.89	1 ± 0.78	0.251
	**5 day**	1.06 ± 0.73	1.24 ± 0.77	1.08 ± 0.81	0.446
	**6 day**	1.04 ± 0.87	1.1 ± 0.98	1.13 ± 0.83	0.881
	**7 day**	1.29 ± 0.79	1.08 ± 0.91	1.02 ± 0.85	0.921
Frequency of using pump **(Mean ± SD)**	**1 day**	6.1 ± 1.37	6.12 ± 1.39	5.61 ± 2.04	0.226
	**2 day**	6.24 ± 1.43	6.46 ± 1.47	5.8 ± 2.13	0.159
	**3 day**	6.06 ± 1.92	6.42 ± 1.76	5.95 ± 2.22	0.469
	**4 day**	6.28 ± 2.02	6.46 ± 1.86	5.76 ± 2.35	0.236
	**5 day**	6.38 ± 2.04	6.53 ± 1.9	5.8 ± 2.4	0.219
	**6 day**	6.6 ± 2.14	6.46 ± 1.89	5.8 ± 2.37	0.157
	**7 day**	6.78 ± 2.2	6.41 ± 1.86	5.69 ± 2.33	**0.046**
Time spent on pump **(min) (Mean ± SD)**	**1 day**	39.9 ± 12.18	39.8 ± 10.28	34.45 ± 14.46	0.056
	**2 day**	40 ± 12.33	40.71 ± 9.94	36.1 ± 14.66	0.068
	**3 day**	42. 7 ± 15.39	41.32 ± 12.49	37.17 ± 15.86	0.164
	**4 day**	45.3 ± 16.42	43.77 ± 12.85	36.52 ± 16.99	**0.015**
	**5 day**	47.2 ± 16.78	43.06 ± 12.11	38.15 ± 17.74	**0.021**
	**6 day**	48.5 ± 17.5	44.59 ± 13.06	37.39 ± 17.69	**0.004**
	**7 day**	48.6 ± 18.01	43.77 ± 13.52	36.95 ± 17.43	**0.003**
Time spent skin-to-skin with the infant **(min) (Mean ± SD)**	**1 day**	18.2 ± 9.4	19.28 ± 10.45	15 ± 9.24	0.088
	**2 day**	20.1 ± 10.71	19.38 ± 9.16	16.19 ± 10.91	0.149
	**3 day**	19.1 ± 11.14	19.79 ± 11.41	16.3 ± 11.71	0.294
	**4 day**	18.2 ± 12.76	19.28 ± 10.75	15.97 ± 11.95	0.388
	**5 day**	16.80 ± 13. 23	19.08 ± 12.69	16.19 ± 12.91	0.516
	**6 day**	17 ± 11.82	17.55 ± 12.46	16.52 ± 11.19	0.914
	**7 day**	17.4 ± 13.21	19.69 ± 12.26	15.11 ± 10.97	0.192
Amount of pumped milk at the end of each day **(cc) (Mean ± SD**	**1 day**	137.9 ± 39.53	131.42 ± 32.38	112.82 ± 47.24	**0.008, 0.007,** 0.06, **0.64**
	**2 day**	151.8 ± 45.15	136.83 ± 38.21	126.3 ± 70.6	0.061
	**3 day**	169.2 ± 66.17	138.26 ± 51.9	125.54 ± 55.18	**0.001, 0.001,0.025,** 0.054
	**4 day**	193.7 ± 78.78	148.77 ± 53.87	130.21 ± 60.67	** < 0.001, < 0.001, 0.002,** 0.35
	**5 day**	220.5 ± 89.65	155 ± 56.77	141.19 ± 69.13	** < 0.001, < 0.001, < 0.001,** 0.63
	**6 day**	244.3 ± 102.21	164.69 ± 63.9	151.41 ± 80. 37	** < 0.001, < 0.001, < 0.001,** 0.72
	**7 day**	268.1 ± 98.53	170.1 ± 66. 69	154.78 ± 82.07	** < 0.001, < 0.001, < 0.001,** 0.69
Infant weight **(gr) (Mean ± SD)**	**0 day**	1262.16 ± 151.48	1263.34 ± 139.1	1219.43 ± 151.39	0.261
	**3 day**	1307.82 ± 162.17	1305.89 ± 140.87	1263.15 ± 155.48	0.287
	**7 day**	1370.12 ± 168.88	1360.91 ± 144.73	1321.3 ± 161.97	0.287

^a,b,c,d^ Repeated measurement test was used for comparison of the three groups and Tukey post hoc test was used for two- by-two comparison of groups

^a^ comparison of the three groups

^b^ comparison of intervention vs control

^c^ comparison of intervention vs placebo

^d^ comparison of placebo vs control

In the between group comparisons, results demonstrated that there was a statistically significant difference in terms of human milk volume in the first, third, fourth, fifth, sixth and seventh days between the three groups (intervention, placebo, and control) (*p* < 0.05) (Table 2).

Results from the analysis of variance with repeated measures for within-group comparisons showed that in the intervention, placebo, and control groups, the mean volume of pumped milk at least one time was statistically significant (*p *< 0.001). Two-by-two comparisons in all times for three groups are presented in Table 3.

**Table 3:** Comparison of within-group of volume of pumped milk in three groups of study

Variables	comparison of the times ^a^	Comparison between seventh and first day	Comparison between seventh and second day	Comparison between seventh and third day	Comparison between seventh and fourth day	Comparison between seventh and fifth day	Comparison between seventh and sixth day
Intervention group	< 0.001	< 0.001	< 0.001	< 0.001	< 0.001	< 0.001	< 0.001
Placebo group	< 0.001	< 0.001	< 0.001	0.003	0.04	1	1
Control group	< 0.001	< 0.001	0.001	0.001	0.033	0.109	1

All of numbers are *P*-value

^**a**^ Repeated measurement test was used for comparison of the times and Bonferroni correction test was used for two-by-two comparison of times in each group separately

There was no statistically significant difference observed in the weight of preterm infants on days 0, 3 and 7 of the study between the three groups of intervention (1370.12 ± 168.88), placebo (1360.91 ± 144.73) and control (1321.3 ± 161.97) (*p* = 0.287) (Table 2). Yet the results in relation to variance with repeated measures for within-group comparisons showed that, in the intervention, placebo and control groups, the mean weight on the seventh day of the intervention was significantly higher than the third day (*p* < 0.001) and day zero (before the intervention) (*p* < 0.001). The mean infant weight on the third day was higher than day zero in all groups (*p* < 0.001).

## Discussion

The purpose of this study was to determine the effect of Pimpinella Anisum herbal tea on human milk volume and preterm infant weight in human populations for the first time. We observed significant differences in milk volume between the three groups. On the first, third, fourth, fifth, sixth and seventh days, the mean volume of pumped milk in the intervention group was significantly higher than the placebo and control groups. The within-group comparison of intervention, placebo, and control groups showed a statistically significant difference between the mean of milk volume before and end of the intervention in each group. These findings in relation to human milk production are congruent with those reported by Hosseinzadeh and colleagues in relation to mice [25], Sallam and colleagues in relation to Egyptian buffaloes [30] and Ying Wang and colleagues in relation to sows [31]. In terms of implications, our findings suggest that Pimpinella Anisum augments human lactation as it does in animal populations. Such augmentation may act as a crucial beneficial factor in preterm infant nutrition worldwide.

Anise causes milk production in animals by stimulating the hormone lactogen (prolactin) [35]. The compounds of Pimpinella Anisum include: anisaldehyde, methylchavicol, eugenol, estragole. It’s most essential oil is anethole, which makes up 93.9% of its content and is considered the main factor of milk production, and the main source of its aroma. This plant is rich in phytoestrogenic compounds and is warm in nature. Structurally, anethole is similar to dopamine and acts as a competitive antagonism in the dopamine receptor. This key mechanism stimulates the release of prolactin and thereby increases milk production [23, 36].

No side effects were reported by participants in our study. Other studies confirming the quasi-estrogenic (phytoestrogenic) effects of Anise have similarly noted an absence of side effects in humans. For example, in reducing the frequency and severity of hot flashes post menopause [37], pain during labor [38] and in improving oligomenorrhea in cases of polycystic ovary syndrome [39]. Our results bring new insights in relation to the use of Anise in human milk production, and further demonstrate safety in its use in human populations.

There was no statistically significant difference observed in terms of preterm infant weight on days 0, 3 and 7 between the intervention, placebo and control groups. The within-group comparison showed a statistically significant difference between the mean of preterm infant weight before and end of the intervention in each group. This contrasts with the findings of Hosseinzadeh et al. (2014), which demonstrated a significant increase in the weight of mice (pups) when compared to control groups [25]. Similarly in contrast with our findings, Anise supplementation has been evidenced elsewhere to improve calf weight gain in line with increased milk volume and feed intake in cows [31]. These differences in findings may be due to our comparatively short intervention period (one week) [40].

All infants participating in the present study were born preterm and were fed through a tube, and only 8.7% of birthing parents participating had a university education. This is important to note as premature infants gain weight more slowly than term infants, and preterm infants along with tube-fed infants, cup-fed infants, and infants with birthing parents having low levels of literacy can weigh even less overall [41]. These factors are important to consider as additional predictors of poor weight gain in preterm infants and may also partially account for some of the findings presented here.

### Strengths and limitations

A key strength of this study is that it is the first to determine the effect of Pimpinella Anisum herbal tea on human milk volume and preterm infant weight in human populations. Limitations include the individual differences in milk production. By allocating the participants in the study groups in a random manner, variable effects may be somewhat mitigated. Due to the prevalence of covid-19 pandemic and the use of home-prepared foods by those hospitalized, it was virtually impossible to standardize the intake of foods that may have a lactating effect. Thus, our findings should be interpreted with caution. However, those who used any additional galactagogues (human milk inducers/enhancers) were excluded from the study, though the supplementation of iron and multivitamins are recommended from the 16th week of pregnancy until three months after giving birth. The present study could not be performed with an intervention duration longer than 7 days, after which time the preterm infant is typically fed human milk directly (rather than being tube fed), and thus the recording of milk production/volume becomes impossible. Future studies of this nature may usefully involve mature infants, allowing for longer intervention periods.

## Conclusion

The results of the present study, along with animal studies around the world demonstrate the effectiveness of Anise in increasing the volume of milk/lactation and the absence of negative side effects. Pimpinella Anisum herbal tea can be used easily, cheaply, and effectively in practice to increase human milk production where appropriate to the benefit of preterm infant nutrition worldwide. Such an intervention would also offer an alternative to any pharmacological supplements considered less favorable.

## Data Availability

The datasets generated and analyzed during the current study are not publicly available due to the confidentiality of information, but they can be available through the corresponding author on reasonable request.
